# Assessing the Impact of Wildlife on Vegetation Cover Change, Northeast Namibia, Based on MODIS Satellite Imagery (2002–2021)

**DOI:** 10.3390/s22114006

**Published:** 2022-05-25

**Authors:** Augustine-Moses Gaavwase Gbagir, Colgar Sisamu Sikopo, Kenneth Kamwi Matengu, Alfred Colpaert

**Affiliations:** 1 Department of Geographical and Historical Studies, University of Eastern Finland, Yliopistokatu 7, 80100 Joensuu, Finland; 2 Ministry of Environment, Forestry and Tourism, Windhoek 13306, Namibia; colgar.sikopo@meft.gov.na; 3 Department of Geography and Sociology, University of Namibia, Windhoek 13301, Namibia; kmatengu@unam.na

**Keywords:** vegetation monitoring, drivers of deforestation, Zambezi region, land degradation, vegetation cover change, wildlife management, TSS-RESTREND, greening and browning, MODIS, Mann–Kendall

## Abstract

Human–wildlife conflict in the Zambezi region of northeast Namibia is well documented, but the impact of wildlife (e.g., elephants) on vegetation cover change has not been adequately addressed. Here, we assessed human–wildlife interaction and impact on vegetation cover change. We analyzed the 250 m MODIS and ERA5 0.25° × 0.25° drone and GPS-collar datasets. We used Time Series Segmented Residual Trends (TSS-RESTREND), Mann–Kendall Test Statistics, Sen’s Slope, ensemble, Kernel Density Estimation (KDE), and Pearson correlation methods. Our results revealed (i) widespread vegetation browning along elephant migration routes and within National Parks, (ii) Pearson correlation (*p*-value = 5.5 × 10^−8^) showed that vegetation browning areas do not sustain high population densities of elephants. Currently, the Zambezi has about 12,008 elephants while these numbers were 1468, 7950, and 5242 in 1989, 1994, and 2005, respectively, (iii) settlements and artificial barriers have a negative impact on wildlife movement, driving vegetation browning, and (iv) vegetation greening was found mostly within communal areas where intensive farming and cattle grazing is a common practice. The findings of this study will serve as a reference for policy and decision makers. Future studies should consider integrating higher resolution multi-platform datasets for detailed micro analysis and mapping of vegetation cover change.

## 1. Introduction

One of the persistent ongoing global environmental challenges is that of land degradation [1,2,3]. Land degradation is quite complex in nature and often involves the inter-play of biophysical, environmental, and socioeconomic factors [4]. There are several scientific debates on what constitutes land degradation but in this study, we adapt the general definition of land degradation by Barbier and Hochard 2018 [5], “as some measurable loss of the biological or economic productivity and complexity of rainfed cropland, irrigated cropland, or range, pasture, forest and woodlands … arising from human activities and habitation patterns”.

Anthropogenic disturbances have been identified as a major driver of land degradation globally [6,7,8,9,10] and are well documented [6,11,12,13,14,15]. The drivers of land degradation are many, complex, and unique across regions [8,13,14,16,17], but these have been categorized as direct and indirect [16,17]. Based on this categorization, the direct causes of land degradation include: (1) infrastructure development (e.g., roads and settlements), (2) expansion of agriculture (e.g., large- and small-scale farming and cattle grazing), and (3) wood extraction (e.g., fuelwood, pole wood, and charcoal production). While the indirect drivers of land degradation include: (a) demographic (e.g., population density, and migration/emigration), (b) economic (e.g., market growth and commercialization, and economic structures), (c) technology (e.g., agro-tech changes), (d) policy and institutional (e.g., formal policies, and property rights), and (e) cultural (e.g., public attitudes and beliefs, and individual and household behavior).

Anthropogenic activities such as wood extraction and conversion of woodland and forests into small and large-scale farming have been a major contributor to land degradation across different geographical regions [15,18]. In the tropics, particularly Africa and in Namibia, agricultural expansion, wood extraction, and infrastructure development are the key drivers of land degradation [9,12,16,17,18].

The impact of land degradation is quite severe in arid, semi-arid, and sub-humid regions [4,19]. Land degradation has a long history in Sub-Saharan Africa and has been well documented and researched [19,20,21]. In Africa, anthropogenic activities, including unsustainable land use practices (e.g., overexploitation of natural vegetation cover), are a major contributor to land degradation, in addition to other natural causes such as droughts [16,22,23]. In a country such as Namibia in south-western Africa, where about 22% of the land area is classified as desert, 70% as arid to semi-arid, and 8% as dry sub-humid [24,25,26], any slight change or modification in the vegetation structure could have adverse effect on the environment, social well-being, and livelihoods of the people [27].

In Namibia, the contribution of anthropogenic activities to the loss of vegetation cover has been well documented [12,24,27,28,29]. The loss of vegetation cover is mostly driven by the conversion of forests and woodland into agricultural farmlands [12,24]. Even though this is the case, the interaction and contribution of wildlife to vegetation cover loss is less understood and needs to be studied in more detail. On the other hand, wildlife damage and human–wildlife conflict is an on-going topic of research and discussion amongst researchers, natural resources managers and various other stakeholders [30,31,32]. This study will focus on one aspect of land degradation: vegetation cover loss and how anthropogenic and wildlife interaction are driving land cover change in the Zambezi region.

Amongst all the human–wildlife conflicts, the African elephant (*Loxodonta africana*) is one of the most significant wildlife species causing structural changes and damage to vegetation [33]. Elephants are herbivores and bulk feeders and require large amounts of food resources to fulfill their nutritional requirements, which they receive from trees, shrubs, and grasses [33,34]. Even though elephants’ consumption of vegetation to meet their dietary needs is natural, overexploitation and mechanical damage becomes destructive, causing vegetation browning and contributing to land degradation (e.g., leaving soils bare) [33,34,35]. The impact of elephants on structural changes in vegetation has been documented by several studies [34,36,37,38,39,40,41,42]. Unlike anthropogenic activities, the impact of elephants on vegetation cover is, to a large extent, confined to locations where elephants exist mostly within protected areas [35,40,43,44]. Anthropogenic restriction of elephant movement and access to space and resources is the main factor driving the browning of vegetation cover by elephants [45,46].

In Namibia, anthropogenic activities are the primary driver of land degradation [12,24], while elephants are mainly responsible for modifying the vegetation structure [47]. As such, we will limit our discussion in this study to the elephant as the major interacting non-human factor contributing to the loss of vegetation cover in Namibia. Additionally, we will use the Zambezi region as a test case, as it is one of the best areas suitable for agriculture in the whole of Namibia and, historically, is home to a wide variety of wildlife (both large and small) [28,48]. The region has the largest savannah woodland cover in Namibia [24,27,28] and is habitat to most elephants in Namibia [30,48]. It is well documented that elephants browse, break, pull and uproot woody species, thus causing structural changes in vegetation cover [44,49,50,51]. The movement of elephants depends on several interrelated factors such as food, water, elevation, density and human settlements [30,49]; they have a home range of from 10 km to more than 8000 km [49]. Consequently, human–wildlife conflicts are a common occurrence in the region [32]. One of the identified reasons for these conflicts is the anthropogenic fragmentation of natural wildlife habitats [30,32,46]. Though this is the case, elephant-induced vegetation cover loss is most likely a secondary cause [51], the primary cause being the limited availability of resources driven by anthropogenic activities [25,46].

One of the solutions to these conflicts has been the establishment of wildlife reserves and national parks and the construction of fences and other barriers to keep wildlife at bay from human settlements [25]. While this has largely worked, the carrying capacity of these wildlife reserves is often not sufficient to sustain large herds of herbivores [25], thus putting pressure on available resources and causing the loss of vegetation cover [25].

Currently, in Namibia, specifically in the Zambezi region, there are projects to combat land degradation [48], conserve wildlife and manage human–wildlife conflicts [51,52,53]. In this study, we will use land degradation to mean the loss of vegetation cover with contextual meaning [54]. Additionally, we will use the terms greening and browning to refer to vegetation increase and decrease, respectively.

Presently, there are multiple satellites that provide datasets that can be used for different research purposes [55,56,57]. Satellite remote sensing is widely used in environmental monitoring, mapping of vegetation, and assessment of different land use and land cover changes [58,59,60,61]. Remote sensing is a popular mode of research as it is the cheapest and most efficient way to assess land use and land cover change [62]. Land use and land cover change assessment is still one of the most important areas of research because of the direct, immediate, and long-term impact of anthropogenic activities on the environment [3,60,63]. Thus, finding long-lasting and sustainable approaches to address land degradation is essential [3,64]. Additionally, it is important to understand that land degradation is contextual in nature and this should be taken into account during discussions [65].

In the Zambezi region, many studies have successfully used satellite remote sensing to assess and map changes in vegetation cover [24,28,29,66]. Although this is the case, to our understanding and best knowledge, assessing and characterizing the impact of wildlife on vegetation cover using remote sensing in the Zambezi region has not been attempted before.

Thus, improving our understanding of the dynamics and impact of wildlife on land degradation within the Zambezi region and beyond is important. Better understanding will provide better insight and tools to improve the management of wildlife and natural land resources in the region. One major challenge of implementing more effective wildlife conservation and natural resource management is continuous access to historical and up-to-date land use and satellite data. Fortunately, the availability of historical satellite remote sensing data and the increasing improvements in analytical software provide opportunities to assess and map changes in vegetation cover and structure. In a 2019 study, the authors successfully applied remote sensing data to characterize regional vegetation cover change in the Zambezi region [24]. In that study, they used eight km resolution Global Inventory Monitoring and Modelling Studies (GIMMS) from the Advanced Very High-Resolution Radiometer (AVHRR) [67]. In the study, only results on a regional scale were obtained due to the coarse resolution of the data [24].

Although higher resolution satellite observations exist, there are drawbacks in using this data, such as (a) the exponentially increasing amount of data result in high computational costs for a long time series, and (b) the low temporal resolution and higher impact of cloudiness (especially in the tropics) [68]. The MODIS 250 m resolution dataset provides over 20 years of continuous global daily imagery, which has been resampled into a monthly NDVI nearly cloudless dataset. This monthly NDVI dataset allows us to use sophisticated geospatial trend analysis techniques.

In this study, we will assess and characterize the vegetation cover change in the last nineteen years (2002–2021).

The specific objectives of this study are to:

(i) Assess the impact of wildlife (elephants and other large herbivores) on the vegetation cover change (greening and browning) in the last 19 years (2002–2021).
(ii) Assess the effects of anthropogenic activities on wildlife migration and vegetation cover change (greening and browning).

To assess the vegetation cover change, we will use historical remote sensing data from the 250-meter Moderate Resolution Imaging Spectroradiometer (MODIS) Terra satellite instrument. We will use time series and geostatistics, as well as geo-spatial analytical methods. Specifically, we will use the Time Series Segmented Residual Trend (TSS-RESTREND) method, which will allow us to separate human-induced land degradation from that caused by natural climatic factors [24,69,70,71]. A similar approach was used by the authors in their 2019 study [24]. Additionally, we will use the Mann–Kendall non-parametric test and Sen’s Slope measure of direction and magnitude of vegetation change [72]. In addition, we will use the Kernel Density Analysis [73] to cluster the elephant GPS tracking data and correlate the results with the trajectory of vegetation cover change.

## 2. Materials and Methods

### 2.1. Study Area

**Figure 1:**
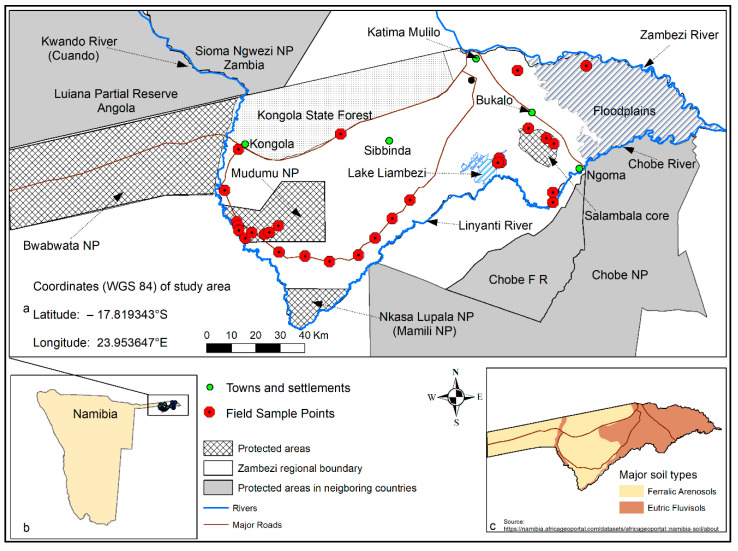
Study area, main roads, rivers, conservation areas, and field work way points for high resolution (2 cm) UAV data in (**a**). In (**b**), the map of Namibia, and (**c**) map of major soil types.

The Zambezi region ((formerly Caprivi Strip) (Figure 1)) is part of the Kavango-Zambezi Transfrontier Conservation Area (KAZA TFCA), stretching across five countries: Namibia, Angola, Zambia, Zimbabwe, and Botswana [48], forming the second largest conservation area in the world [51]. The land area of the Zambezi region is 14,785 km^2^ [51], with a total population of 98,849 (2011 Census) [24]. Most of the vegetation in the region is woodland savanna and open grasslands [27,28,51]. The region contains three large National Parks, Bwabwata, Mudumu and Nkasa Lupala (formerly Mamili) [48,51]. Conservancies in the region include: Kwandu, Mayuni, Salambala, Sibbinda and Linyanti [25,29]. A conservancy is a legally defined area set aside and managed by local communities who have rights to live within, use, and manage wildlife and other natural resources for personal and tourism purposes (including trophy hunting) [74,75]. The region is an important migratory route and home to a high density of elephants [25,76], buffalos, and antelopes [48]. Additionally, the region is an important agricultural area, due to good soils and high rainfall [77,78].The major soil types in the region are poor ferralic arenosols containing high iron contents and fertile eutric fluvisols with high base saturation [77,78]. The yearly amount of rainfall in the region is the highest in Namibia (500–700 mm/year) [77,78] when compared to the national mean of <250 mm/year [19] and <50 mm/year in the southwestern and coastal areas [77]. The wet season in the region starts in November and ends in April. The average summer and winter temperatures in the region are 35 °C and 5 °C, respectively [78]. The region has a yearly evaporation rate of about 2500 mm [78]. The region shares common borders with Angola, Botswana, Zimbabwe, and Zambia [48]. The perennial Kwando (Cuando) River flows along the border between Angola and Zambia through the Zambezi region (with Bwabwata National Park on the west and the Mudumu National Park and the six conservancies on the east) south towards the swampy areas around Nkasa Lupala National Park. East of Nkasa Lupala is the Linyanti River that flows east through the seasonal Lake Liambezi into the Chobe River. The Chobe River flows eastward into the perennial Zambezi River, one of Africa’s major and longest river systems. The Zambezi River flows from Zambia and forms the border between Zambia and Namibia in the Zambezi region.

### 2.2. Satellite and UAV Field Data and Image Pre-Processing

We downloaded and processed the monthly 250 m Moderate Resolution Imaging Spectroradiometer (MODIS) Terra satellite instrument NDVI datasets. We resampled the NDVI index to a common 250 m grid (UTM-35S). Because the 2001 data set is incomplete, we used only raster images from 2002 to 2021.

The time series for precipitation and temperature data were monthly ERA5, available from 1979 (ERA documentation). The monthly 0.25 × 0.25 degree resolution data was downloaded from 1999 to 2021 (https://cds.climate.copernicus.eu (accessed on 6 May 2022)). Both temperature and precipitation were resampled to a common 250 m grid and re-projected to the UTM reference system. The temperature and precipitation data must start two years before the NDVI time series because this information is required by the processing algorithms during analysis to calculate the maximum rainfall accumulation months. Finally, we used 265 gridded monthly temperature and precipitation raster layers.

### 2.3. Field Sample Locations and Elephant Tracking Data

During our field work, we used a hand-held GPS instrument [(Garmin GPSMAP 62ST), Garmin Finland] to collect the latitudes and longitudes of sample points. At each sample point (Figure 1), we recorded the location coordinates and geographical name. We used a DJI Mavic Pro Platinum drone to document the vegetation characteristics by taking aerial photos and videos at every sample point. At each sample location, we flew the drone at a height of 40–90 m and recorded a 360° view of the surrounding vegetation (pictures and videos). 

The Government of the Republic of Namibia (Ministry of Environment Tourism and Forestry) provided the elephant tracking data (2010–2020). These data are a transboundary hourly GPS-collar tracking dataset, covering Namibia, Botswana, Angola, and Zambia. The data consisted of 31 individual elephants over a period of eight (8) years (2010–2020). The GPS-collared elephant data were collected by the Africa Wildlife Tracking company (https://awt.co.za/ (accessed on 6 May 2022)), based in Pretoria, South Africa. The GPS collars were put on the elephants by first using a tranquilizer dart from a helicopter to immobilize them. The brand of GPS collar used was the Iridium Satellite (IR-Sat) that collects and transmits continuous near real-time data. The data transmission and receiver of the IR-Sat covers a few hundred meters to multiple kilometers [79]. Table 1 presents a breakdown of the number individual elephants tracked and during which period. We downloaded additional crowd sourced wildlife observations, elephant and buffalo observations (one kilometer grid), using the Monad (1 km × 1 km) reference grid data from the Environmental Information Service Namibia (http://www.the-eis.com (accessed on 6 May 2022)).

### 2.4. Data Analysis

To assess the vegetation changes, browning (decrease) or greening (increase), we used the Time Series Segmentation and Residual Trend analysis (TSS-RESTREND) method [69] to perform a pixel wise analysis. To achieve this, we created and used an R-script that iterates over each pixel across the image stack of the complete the time series. TSS-RESTREND is an improved method of the Residual Trends algorithm (RESTREND) [71] that incorporates the functionalities of Break For Additive Season and Trend (BFAST) algorithm [80,81] to look for break points in the time series.

RESTREND uses an Ordinary Least Squares Linear Regression model, fitted on the residual and time [1,24]. The equation is of the form:

(1) $$y_{i}=\beta_{0}+\beta_{1}x,$$

where x is time in years, β_0_ is the intercept and β_1_ is the slope.

BFAST fits a linear piecewise harmonic model using the ordinary least squares moving sum (OLS-MOSUM) to test for structural changes within time series data [24,82].

The decomposition model takes the following form:

(2) $$Y_{t}=T_{t}+S_{t}+e_{t},$$

where Y_t_ is the original observed data (TS) at time t, T_t_ is the trend, S_t_ is the seasonal, and e_t_ is the remaining unexplained variation within the TS, respectively [24,80].

TSS-RESTREND fits a multivariate regression between the VPR-Residual (vegetation precipitation) and a dummy variable (0 before a break point and 1 after). The model is of the form:

(3) $$y_{i}=\beta_{0}+\beta_{1}x+{\;\beta }_{2}z_{i}+{\;\beta }_{3}x_{i}z_{i}$$

where x is time in years, β_0_ is the intercept, β_1_ is the slope, β_2_ is the offset at breakpoint position, β_3_ is the change in slope at the breakpoint position and z is the dummy variable (0 or 1) [24,69].

In addition, we performed a pixel wise Mann–Kendall statistics test of the NDVI time series to determine the trend of total vegetation change in the Zambezi, and the Sen’s Slope to determine the magnitude of the change [72]. Mann–Kendall is a non-parametric test and does not rely on a particular data distribution but rather on the relative magnitude of the sample data [83,84].

The Mann–Kendall statistics is of the form:

(4) $$S=\overset{n-1}{\underset{k=1}{\sum }}\overset{n}{\underset{j=k+1}{\sum }}\mathrm{sign}\left(x_{j}-{\;x}_{k}\right)$$

where:

(5) $$\mathrm{sign}\left(x_{j}-{\;x}_{k}\right)=\{\;\begin{array}{c} 1\;if\;x_{j}-x_{k}>0\\ 0\;if\;x_{j}-x_{k}=0\\ -1\;if\;x_{j}-x_{k}<0 \end{array}$$

x_j_ and x_k_ are the annual values in years j and k, respectively [29].

The non-parametric Sens Slope time series analysis was performed using the same pixel-wise moving window method to obtain the linear rate of change in the time series. During all pixel-wise analysis (TSS.RESTREND, RESTREND, Mann–Kendall and Sen’s slope), we used a *p*-value parameter of 1 during the analysis, thus we obtained the change in every pixel, irrespective of the *p*-value. We took this approach because it provides for a synoptic spatial overview, showing gradual changes between distinct areas of significant degradation and vegetation increase, and areas of no change; the latter is associated with non-significant *p*-values. We observed that the changes that the algorithm interpreted as non-significant contain important information, e.g., areas of no change. This approach also provides a much more homogeneous and easier to understand cartographic map product. Although the general result of the different methods conforms very well, local differences are noticeable when comparing the results of different algorithms, therefore we made an additional ensemble analysis by combining the TSS.RESTREND, RETREND and Mann–Kendall algorithms and calculating the mean value of the results.

**Figure 2:**
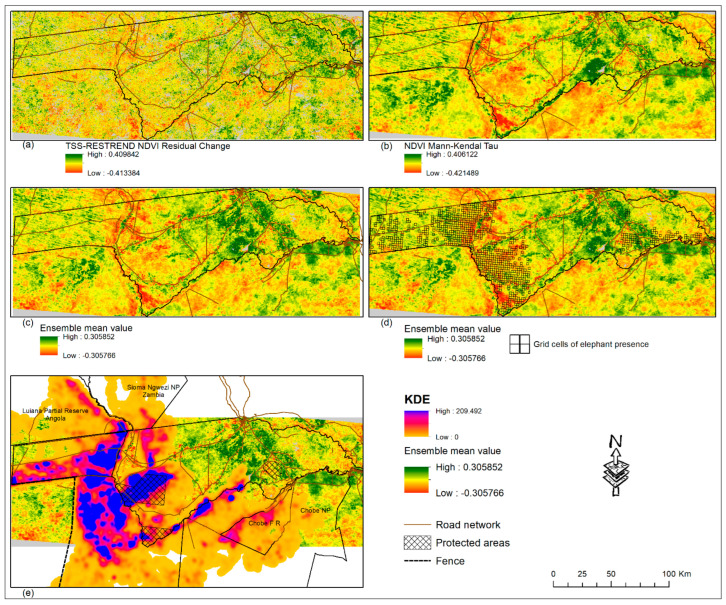
(**a**–**e**) show the output of the TSS.RESTREND, Mann–Kendall, ensemble, and kernel density estimate (KDE) analysis over the whole Zambesi region. In (**a**), the Residual Change of the TSS.RESTREND and (**b**) is the Mann–Kendall Tau. In (**c**) is the ensemble of the mean values of RESTREND, TSS.RESTREND, (**d**) the spatial distribution of elephant sightings overlayed over the ensemble mean value from (**b**) above and (**e**) the overlay of the Kernel Density Estimate (KDE), and Mann-Kendal Tau from (**b**) above. No data and background values are displayed as grey.

**Figure 3:**
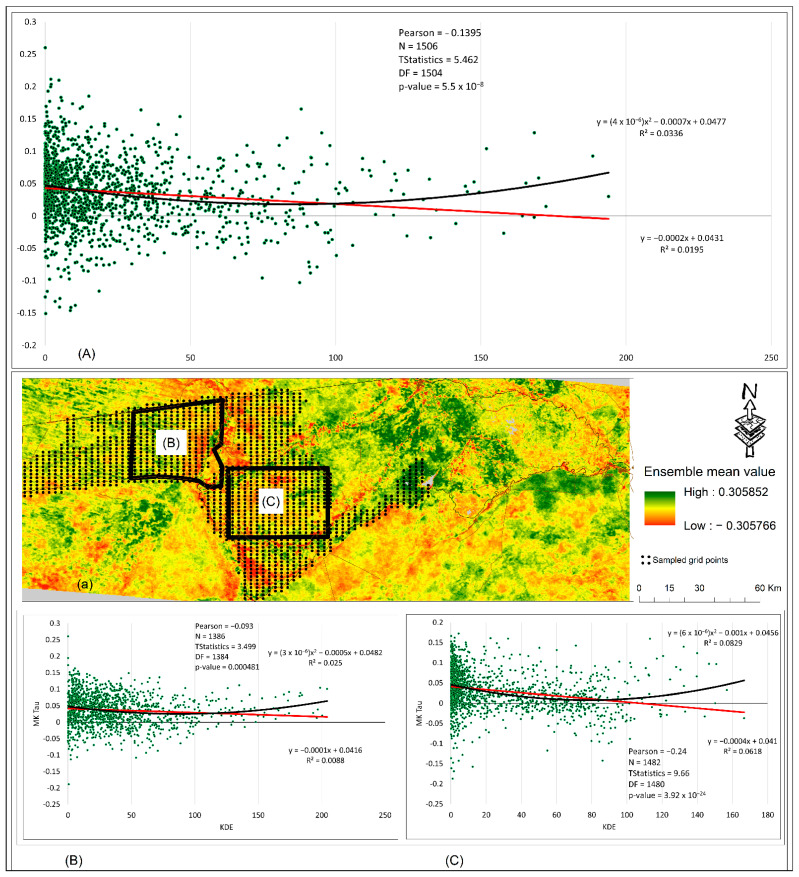
Shows the locations of the sampled grid points in the Zambezi region of the Kernel Density Estimate and the ensemble. The grid points are overlayed on the ensemble (a) used previously in Figure 2. The results of the Pearson correlation in (**A**) correspond to all the sampled points in the Zambezi while (**B**), and (**C**) correspond to the sub-sampled areas (black polygons) above.

We computed a Kernel Density Estimation (KDE) of the elephant tracking point data in ArcMap 10.5.1. We then used the KDE and the ensemble mean to calculate a simple Pearson correlation analysis between the presence of elephants and vegetation changes. Before calculating the Pearson correlation, we used ArcMap 10.5.4 to create a grid of 1600 points over the whole Zambezi area where elephants are present (Figure 2 and Figure 3). Additionally, we created an additional l600 points in two sub-sample grids, west of the Kwando River in Bwabwata park and around the Mudumu park (Figure 3B,C). We then extracted the values of the ensemble mean at these points and we excluded data points where the KDE was zero (no presence). We were left with 1506 points 1386 points in sub-sample locations one (Bwabwata) and 1482 points in two (Mudumu). We then used these points to compute a simple Pearson correlation in Microsoft Excel (version 2202).

To validate our results, we compared the UAV data we collected during our 2019 field trip with the outcome of the time series analysis. We used R (R Core Team, 2022) and ArcMap (version 10.5.4) for data analysis and to produce graphics. The R-code was run on the cPouta cloud services of CSC using 24 and 48 cores Ubuntu Virtual Machines (https://research.csc.fi (accessed on 6 May 2022)). We implemented Google Earth Pro for visual interpretation and verification of results.

## 3. Results

### Pattern of Vegetation Trend: 2002–2021

**Figure 4:**
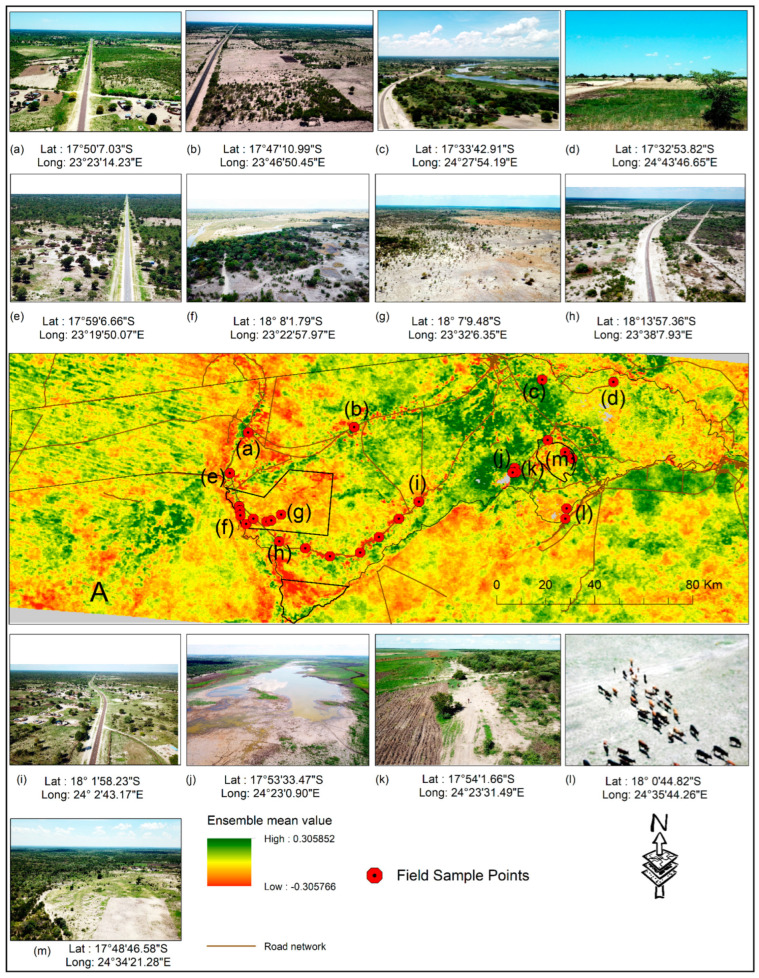
Shows aerial images from our 2019 field survey in the Zambezi region (**a**–**m**). In (A), the labels (**a**–**m**) correspond to the images shown with their respective GPS coordinates at the sample locations (red circle with black dot in the middle). Additionally, in (A), we use the same ensemble as in Figure 2b above for reference purposes.

Based on remote sensing and GIS data, this study analyzed the human–wildlife interaction in the Zambezi region. Figure 2 shows the pixel-by-pixel vegetation change pattern in the Zambezi region during the period 2002–2020. The TSS-RESTREND residual change (Figure 2a) highlights a mixed pattern of positive (greening) and negative (browning) vegetation changes. Similarly, the ensemble and Mann–Kendall Tau (Figure 2b,c) both clearly show a mixed pattern of positive and negative values. The positive and negative pixel values indicate vegetation change increase and decrease, respectively. The observed negative pattern of the TSS-RESTREND and RESTREND is attributed to factors other than climatic aspects because the variability associated with climate was removed during the analysis. Most of the browning pixels (land degradation) are along the Kwando River (Figure 2a, Figure 3 and Figure 4A), which is a major migration route for wildlife, specifically elephants [25]. On the eastern part of the Kwando River are two National Parks Mudumu and Nkasa Lupala, and six conservancies (Kwandu, Mayuni, Mashi, Balyerwa, Wuparo, and Malengalenga), (Figure 1, Figure 2a and Figure 3) [25]. On the western part of the Kwando River is the Bwabwata National Park, a part of the home range for large herds of elephants [32], and also contains a buffalo core area [25]. Most of the vegetation browning we observed was also taking place within Mudumu and Nkasa Lupala National Parks (Figure 2). Within Bwabwata National Park (west of the Kwando River), browning is mainly close to and along the Kwando River, while elsewhere most of the pixels show greening (Figure 2).

Figure 3 shows the Pearson correlation between the Mann–Kendall Tau and the Kernel Density Estimate of the elephant data. Over the whole Zambezi (Figure 3A), as well as in the western (Figure 3B) and eastern areas (Figure 3C), there is a clear negative trend (browning) indicated by the red straight line. In the whole Zambezi area (Figure 3A) and in both sub-sampled locations (Figure 3B,C), the negative trend (browning) is significant (*p*-values = 5.5 × 10^−8^, 0.0005, and 3.93 × 10^−24^). Additionally, the results (Figure 3A–C) show that as the density of elephants decreases away from the KDE core areas, vegetation greening begins to occur (black polynomial line, Figure 3A–C). It is noteworthy that the polynomial line increase related to high KDE densities probably indicates that large herds are attracted to abundant vegetation resources. Moving further to the eastern part, we observed browning around and within the Salambala core area, located east of Lake Liambezi (Figure 2c). The Salambala core area is also home to elephant herds (Figure 2e). In addition, we also observed browning close to and around roads (Figure 2, Figure 3 and Figure 4). These roads are locations of high-density human settlements where cattle grazing and extensive agricultural activities are a common practice. On the other hand, we also observed some relatively high greening, mostly around Lake Liambezi, communal areas, as well as some locations of the floodplains (Figure 2, Figure 3 and Figure 4).

The browning pattern we observed is not only confined to the Zambezi region but extends across the border into neighboring countries (Figure 2). For example, the browning along the Kwando River continues across the Namibian border into Luina Partial Reserve (Angola) and Sioma Ngwwezi National Park (Zambia). We also observed a similar pattern across the border into Botswana, where we can see a clear difference along the 135 km veterinary fence which was constructed between 1991 and 1997 [25], (Figure 2c,e). On the eastern side of the fence, we observed high levels of degradation, while to the west we see relatively high greening values (Figure 2c).

**Figure 5:**
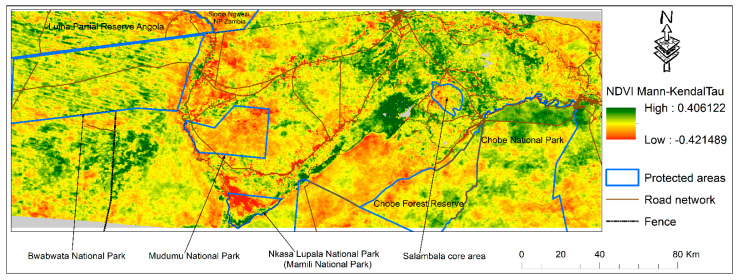
Additional resources.

We also ran the Mann–Kendall test on temperature and precipitation but did not see any significant trend, so the result was not shown here. An enlarged graphics of the Mann–Kendall NDVI Tau is shown in Figure 5 (Additional resources).

## 4. Discussion

### Potential Impact of Wildlife on Vegetation Cover and Land Degradation: 2002–2021

This study found that human–wildlife interaction is driving vegetation cover change in the Zambesi region. Specifically, anthropogenic restriction of space and resources for wildlife is driving the observed accelerated vegetation removal by large wildlife herbivores, in this case elephants. Consequently, this interaction is a potential contributor to land degradation in the Zambezi region. Previous studies in the Zambezi region by Gbagir et al. 2019 [24] revealed that land degradation is driven by the interaction of multiple direct and indirect factors. These factors include: demographic [24,27], ecological (e.g., floods) [27,85], and environmental factors (e.g., topography) [24,28]. Specifically, subsistence farming, infrastructure expansion (e.g., roads), including settlements, and legal and illegal wood extraction for firewood were identified as the main drivers of land degradation in the region [24]. However, the studies by Gbagir et al. 2019 [24] were not able to clearly establish more specific causes for land degradation due to the nature of the data used [24].

In the present study, a more detailed pattern and trend of vegetation cover change was revealed and the impact of wildlife on land degradation was clearly established. The contribution of wildlife on land degradation in the region corresponds to previous base line studies and ongoing statistics from the region. Reports indicate that elephant populations in the Zambezi region are stable or growing [51,86]. In addition to elephants, other wildlife populations, e.g., buffalo, are present (Figure 2d), but since elephants clearly damage trees, our results and discussion are focused on these.

Baseline studies on the presence of elephants in Namibia were carried out in the early 1980s and 1990s [32,87]. Based on these studies, several subsequent surveys have shown that the population of elephants has been increasing steadily in Namibia [25,48] from 600 to 1000 in 1934 to 22,754 in 2016 [48]. Probably the elephant population can be assumed to be even larger in 2022. The Zambezi region (Namibian KAZA) hosts most of the elephants in Namibia and is the most important migration route [30,48]. Reports show that the number of elephants within the Zambezi region has more than tripled since 1995/1996 [48]. A current estimate of elephants in the region is reported as 12008 [88]. According to Chase and Griffin (2009) [25], the elephant population in the Zambezi region was 1468 in 1989, while in 1994, these figures were reported as 5804 [25], 7950 and 5556 [87,88].The differences between the figures are mainly due to the different sampling techniques used in those studies [25,88]. By 1998, these figures were down from 5804 to 4576 [25], but in 2005 the numbers had again increased to 5242 [89]. The decline in the elephant population was thought to be the result of the civil unrest in Angola [25,89] and the construction of the veterinary fence between Namibia and Botswana in western Zambezi [25,48,76], consequently restricting and cutting off the migration of wildlife (elephants, buffalos, wildebeests, zebras, etc.) [25,48,76].

Most of the browning we observed was along the Kwando River (Figure 2), an important migration corridor for elephants [32]. The negative impact (browning) on the surrounding vegetation is clearly visible compared to elsewhere in the Zambezi region, as elephants browse on and de-back and break down trees, causing structural changes to the surrounding vegetation [47,90]. The Kwando River and the Mudumu and Nkasa Lupala parks contain medium to high numbers of elephants [32]. The studies by O’Connell-Rodwell et al. 2000 [32] put the number of elephants present on the western Kwando River as 3000, while 400 and 600 were reported for Mudumu and Nkasa Lupala, respectively. Within Bwabwata national park (west of the Kwando River), browning is limited to the riverbanks and migration route, while elsewhere, there is less indication of high levels of browning. We also observed a similar pattern of browning within the Salambala core area, while most of the greening is occurring elsewhere in the area (Figure 2c). A 2019 survey of elephants reported the current population in the Salambala conservancy as 507 [88].

Other important factors contributing to land degradation in the Zambezi region are of anthropogenic origin, as has been established by previous studies [24]. However, in this study, we now see clearly how these anthropogenic activities have contributed to wildlife-induced vegetation browning in the region. The expansion of settlements and roads and the construction of artificial barriers (e.g., fences) has diminished the habitat of elephants and reduced their access to food and water resources [25,45,47,91]. As a result, more pressure is put on the remaining resources, propagating vegetation browning in the region [25,45,47,91]. Additionally, the shrinking of elephants’ habitats has modified the behavioral pattern of these animals [46] and increased human–wildlife conflicts [53,66]. Most of the visible browning was observed within the protected areas (e.g., Mudumu, Nkasa Lupala, Salambala core) due to the high density of elephants concentrated within small restricted areas. Restricting the habitat of wildlife, specifically elephants, has impacted and contributed to the observed browning [45,91]. The Pearson correlation results (Figure 3A,B) also confirmed that the concentration of elephants within a certain restricted area was contributing to the observed gradual browning. However, the right side of the curve (Figure 3A,B) show that areas of vegetation browning do not sustain high animal populations; hence, the curve rises as the green areas attract large herds, thus indicating that the available space and resources may be beyond the carrying capacity of the current number of elephants with the protected areas. Additionally, taking into consideration that the arenosols soils are poor in nutrients [77,78], any slight modification in the vegetation cover will have a visible impact, which in this case is browning.

In addition to the elephant populations in this area, along the Kwando River there are also six conservancies (Kwandu, Mayuni, Mashi, Balyerwa, Wuparo, and Malengalenga) [25]. The presence of these settlements has given rise to clearing of land for farming and cattle grazing and increases in the road network. The presence of human settlements increases human–wildlife competition for land resources, making these areas hotspots for human–wildlife conflicts. Just like the six conservancies along the Kwando River, the Salambala core is also surrounded by several villages where farming and cattle grazing is a common practice [24,29,92,93], which could explain the high levels of vegetation browning (Figure 2c).

Apart from browning, there is also greening within the Zambezi region, most of the greening is occurring within the communal areas of Lake Liambezi and the Chobe River floodplains farther east. These greening areas have high human population densities and are locations of intensive farming and cattle grazing [48]. This we were able to verify during our field visit in December 2019 (Figure 4).

The greening of the Lake Liambezi is mostly due to the present drying (Figure 4j) which opens land for vegetation growth, farming, and grazing activities (Figure 4k). Large numbers of cattle are grazing on the eastern floodplains (Figure 4l) [48]. Recent reports in 2019 estimated the total number of cattle in the Zambezi region to be 135,878 animals [88].

The pattern of vegetation browning observed along the Kwando migratory route continues into neighboring Zambia (Sioma Ngwezi National Park) and Angola (Liuana Partial Reserve) (Figure 1 and Figure 2). Additionally, this pattern of vegetation browning applies to the southern border with Botswana around the Chobe National Park (Figure 2, Figure 3 and Figure 4). The Chobe National Park is where the majority of the 200,000 migratory elephant population is located [24,94,95]. Furthermore, it is quite clear that greening is mainly on the opposite side (south) of the northern buffalo fence, where access by elephants is restricted [9,35] (Figure 2c,e) [59].

Similar studies elsewhere have also linked elephants to the loss of vegetation in protected areas. Examples include: Samburu and Buffalo Springs National Reserves [39], Aberdare National Park [44] in Kenya, Addo Elephant National Park, Eastern Cape, South Africa [38], and the Serengeti National Park, Tanzania [40,42]. We anticipate that the results of this study will provide increased understanding of the interaction between wildlife and land degradation in the Zambezi region. This new additional information could potentially improve and inform policy formulation and decision-making regarding wildlife and natural resources conservation and management in the region and elsewhere in Namibia.

Future studies should consider detailed and micro-analysis, classification and mapping of vegetation cover change by combining and integrating higher resolution remote sensing datasets [56,61,63,96,97,98]. This form of information will provide even better data to improve the current integrated sustainable land use and management practices.

## 5. Conclusions

This study assessed the impact of wildlife populations, specifically elephants, on vegetation browning in the Zambezi region during the last 19 years (2002–2021). Our analysis reveals that vegetation browning is mostly in locations with a high density of elephants. Most of the browning is along the migration corridor of elephants within national parks and conservation areas as a result of exclusion and harassment in areas with human settlements. Obviously, brown vegetation areas do not sustain high population densities of animals.

We also found that the expansion of settlements and the construction of artificial barriers (e.g., fences) has affected the movement and migration pattern (behavior) of wildlife populations, specifically elephants, in the region, which has led to the concentration of game animals within confined national parks.

Furthermore, the limited amount of space and resources for wildlife populations could potentially be a major factor contributing to vegetation browning in the region. This assumption is supported by the high incidence of ongoing human–wildlife conflicts within the region. On the other hand, our study found that most of the greening was occurring in areas with intensive farming; for example, around the shrinking Lake Liambezi, and within communal areas.

## Figures and Tables

**Table 1:** The tracked elephant data used in this study. The periods correspond to the 12 calendar months of the year. The total length of tracking period (in months) is shown in brackets.

Year	No. of Individual Elephants Tracked	Period
2010	8	10–12 (3)
2011	7	1–12 (12)
2012	7	1–8 (8)
2016	5	3–12 (8)
2017	13	1–12 (12)
2018	20	1–12 (12)
2019	16	1–12 (12)
2020	6	1–12 (12)

## Data Availability

Not applicable.
